# Exploratory study to identify mechanical factors that may contribute to toe dactylitis in patients with psoriatic arthritis

**DOI:** 10.1186/1757-1146-7-S2-A8

**Published:** 2014-11-14

**Authors:** Richard Wilkins, Heidi Siddle, Anthony Redmond, Philip Helliwell

**Affiliations:** 1 Section of Clinical Biomechanics and Physical Medicine, University of Leeds, Leeds, UK

## Background

Dactylitis (sausage digit) is one of the most commonly reported features of psoriatic arthritis (PsA), the second most common inflammatory arthritis after rheumatoid arthritis (RA). It has been hypothesised that dactylitis is a functional enthesitis at the proximal interphalangeal joints (hands and feet), causing multiple pathologies to varying levels of severity. Dactylitis results in synovitis, tenosynovitis, bone and soft tissue oedema to the digit, described as tender and non-tender dactylitis. Trauma and physical insult to the digit have been suggested as a possible cause. The aim of this study was to explore the mechanical factors that may contribute to toe dactylitis in patients with PsA.

## Methods

Twelve participants with PsA and a history of dactylitis (group i), 12 participants with PsA and no history of dactylitis (group ii), and a control group of 12 participants (group iii) were recruited. Plantar pressure measurements were undertaken barefoot using the Novel Emed-SF pressure system, and in footwear using the Pedar in-shoe pressure measurement system. Peak plantar pressure and pressure time integral were analysed at the most common and second most common sites of dactylitis reported in the foot; 4th and 2nd toes, and 4th and 2nd metatarsophalangeal joints (MTP) of the left foot. Temporal and spatial parameters of gait were collected using the GAITRite system. Patient reported impairment and footwear (FISAP), and activity limitation and participant restriction (FISIF) were reported using the Foot Impact Scale for Rheumatoid Arthritis (FIS-RA).

## Results

Thirty-six participants took part in the study. PsA patients had a mean disease duration of 4.58 years in both groups, a mean FISIF score of 7.16 in group i and 6.83 in group ii compared to 0.41 in the control group, and a mean FISAP score of 8.75 in group i and 5.75 in group ii compared to 0.16 in the control group. ANOVA analysis and subsequent post-hoc testing using Games-Howell test yielded significance in FIS-RA mean scores. In both domains of the FIS-RA there was a significant difference between both PsA groups compared to the control group; PsA group i p = 0.00 and PsA group ii p = 0.00 in the FISIF domain, PsA group i p= 0.03 and PsA group ii p= 0.05 in the FISAP domain. Mean plantar pressures measurements barefoot in-shoe at the 2nd and 4th toe, and 2nd and 4th MTP joints were not significant between groups. No significant differences were reported in spatial and temporal parameters of gait between groups.

## Conclusions

This is the first exploratory study to investigate the mechanical factors that may cause dactylitis in patients with PsA. FIS-RA scores indicate PsA patients have significant limitations compared to controls, but a history of dactylitis does not worsen patient reported outcomes. Although no significant differences could be reported in plantar pressure data or gait variables, the study was underpowered. A subsequent power calculation indicates that 60 participants per group would be needed to power a larger study. Exploration of shear and friction in the forefoot may provide insight for a biomechanical trigger to dactylitis.

